# Rabies, with a Report of Two Recent Outbreaks

**Published:** 1898-11

**Authors:** A. B. Kelly

**Affiliations:** Cohoes, N. Y.


### RABIES, WITH A REPORT OF TWO RECENT OUTBREAKS.

By A. B. Kelly, D.V.M.,
COHOES, N. Y.

(Concluded from page 666.)

*Diagnosis.* After searching the history of a case thoroughly, and carefully considering the objective symptoms, an expert can generally diagnose a case of rabies, although many cases will demand the animal-inoculation test. We may confound this affection with hysteria, tetanus, encephalitis, lead-poisoning, tumors of the plexus, spasmodic sore-throat, several parasitic diseases, and lyssaphobia. In all these cases the history is lacking—the patient has not received a bite, there has not been any strange dog about, the disease is not known in the locality, the attack is sudden, the victim is not vicious, and animal inoculation gives negative results.

The excitement occasioned by the presence of a dog is very characteristic.

A case of pseudophobia is diagnosed by the length of the period of incubation, the absence of some of the most characteristic symptoms, owing to the person's lack of knowledge of the malady, by the mildness of the hyperæsthesia, and by the readiness with which the disease yields to mental treatment, such as the mad-stone and reasoning.

*Method of diagnosing rabies by animal inoculation.*[10] Rabbits or guinea-pigs are commonly used, as they are easy to handle and quite inexpensive. Have an assistant place the animal under the effects of a general anæsthetic (ether generally preferred); cut the hair off of the section between the eyes and ears, wash with soap and water, followed by a disinfectant. During this time the operator should sterilize all the instruments to be used, and thoroughly cleanse and disinfect his hands. When complete anæsthesia is produced, make a longitudinal incision in the skin and subcutaneous tissue, and use a speculum to widen the aperture; then make a crucial incision in the periosteum on one side of the median line, to avoid hemorrhage from the longitudinal sinus; push aside the four flaps and insert a small trephine, removing a small piece of bone, exposing the dura mater, and with a hypodermic syringe inject one or more drops of a suspension in water of the suspected brain beneath the dura, replace the periosteum; suture the skin, disinfect and return the animal to a cage. If the inoculation has been successful, the animal will soon recover from the effects of the operation, and will generally eat heartily the second meal thereafter.

To prepare the suspected brain for injection remove it with aseptic precautions, place a small piece of it in a sterilized mortar, and grind finely with a few cubic centimetres of sterilized water or bouillon.

After recovery from the operation the animal should remain well until the specific symptoms begin to appear, usually from fifteen to thirty days after the inoculation. Rabbits rarely become furious, but at times they are rather nervous for one or two days prior to the paralysis. There appears to be marked hyperæsthesia. Usually the first indication is monoplegia or paraplegia, but soon the whole animal is affected, and lies prostrated until relieved by death, which usually occurs within twenty-four hours after the complete paralysis sets in. Although all voluntary movement is lost, the reflex action may be noticed until within a few hours of death. During the period of incubation the temperature is normal until near time for the first symptoms to appear; then there is a rise of 1° or 2° F., which lasts for one or two days, and then suddenly drops to subnormal, where it remains until death.

The differential diagnosis, in this way, is not very difficult. If death were due to septicæmia there would be marked lesions in the brain and viscera, and by bacteriological examination of the blood the microörganisms would be found. If death were due to mechanical injury it would occur very soon after the inoculation, or, at least, the paralysis would be preceded by a stage of disability. A paralytic stage precedes death from various pathogenic germs, but the period is short, and you would invariably have the symptoms and lesions of the specific disease. Therefore, this method requires that the inoculated animals remain well for a considerable length of time after the subdural injection and before the paralysis appears; the wound in the head should be healed perfectly, with no abscess-formation; the meninges should be free from exudates, and the brain should appear perfectly normal, except in rare cases there may be a slight congestion. The liver may be deeply reddened, and the gastric mucosa may show dark patches, indicating disintegrated hemorrhagic areas. The intense rigor mortis following a death from rabies is very important.

*Treatment and Prevention.* The treatment is almost exclusively prophylactic, as very few cases of recovery are recorded where the disease has developed. In a country where rabies exists the bite of any carnivorous animal should be treated as though the assailant were known to be rabid, as any one of this species that has been allowed to run at large may harbor an attenuated virus which would rapidly propagate in the victim.

Therefore one should, if possible, thoroughly cauterize such a wound with some active chemical caustic or the actual cautery. If the wound is on a limb, and no caustics can be obtained, he should, if possible, apply some form of a tourniquet to arrest the circulation, and drink warm liquids to prevent absorption. Suction is important, this being accomplished either through a tube or by the lips, the mouth being frequently washed out by some disinfectant. When using solid caustics, such as nitrate of silver, chloride of zinc, caustic potash, or sulphate of copper or iron, it is necessary to apply them to all parts of the wound, and if a fascia prevents this, it should be severed. If liquid caustic, such as the mineral acids, be used, a pledget of cotton on a forceps or stick will generally suffice. Probably the thermo-cautery is the best, as by this you can reach any part of the wound, and it does not expose any new surface without first cauterizing it. If a tourniquet has been applied, it should not be removed until the wound has been thoroughly cauterized. One should not omit the cauterization on account of a delay after the bite. Some advise excision of the parts, but in doing this, strict antiseptic principles should be adopted. It may be necessary to amputate a member, if its tissues are very badly lacerated; but in the lower animals this is only resorted to when the wound is in the ear or tail, to prevent absorption of the poison.

A wound should not be neglected because it is small, as some of the smallest are the most serious. Even if the flesh is not broken the part and clothes covering it should be thoroughly disinfected, as the virus may gain entrance through some other laceration.

The victim may be in great pain caused by the wound, in which case it may be necessary to give morphine, chloral, bromide of potassium, or calabar bean hypodermically; suppositories of morphine may also be used. Any symptom appearing during the attack should be treated as such, and great care must be given to the general health. When once well established the disease is incurable, the only treatment possible being palliative, the victim being placed in a darkened room, with not more than two careful attendants. By the local application of cocaine to the throat a patient may be enabled to take food; otherwise nutriment must be administered in the form of an enema.

One of the best-known treatments is that devised by M. Pasteur; it consists of a series of subcutaneous injections of the rabid virus, the potency of which is so regulated by the following method that a stronger virus may be injected each succeeding day until the system is so educated that it prepares an antitoxin which will combat the virulent action of the virus of rabies.

To prepare this virus he inoculated rabbits subdurally, one from another, until the disease developed in six or seven days after the inoculation; the virus then having attained its greatest potency is called the "fixed virus." These last rabbits are killed, and aseptic sections of their cords are preserved *in vitro*, in free but aseptic air (which has been dried by contact with caustic potash) for periods varying from one to fourteen days. The virulence lessens day by day, so that the fourteenth-day product can be inoculated with perfect safety.

The injections are made in the hypochondriac region, as the cellular tissue is loose and very absorbent in this part.

He advises two forms of treatment, namely, a simple form, for those bitten through clothing, and an "intensive" form for those bitten on the face, throat, or hands.

The simple treatment is as follows:

| | | | | | | | | | | | |
|---|---|---|---|---|---|---|---|---|---|---|---|
| 1st | day | two 3 | cc. | doses | of | solutions of | 14th | and | 13th | -day | brains. |
| 2d | " | two 3 | " | " | " | " | 12th | " | 11th | " | " |
| 3d | " | two 3 | " | " | " | " | 10th | " | 9th | " | " |
| 4th | " | one 3 | " | dose | " | " | 7th | | | " | " |
| 5th | " | two 2 | " | doses | " | " | 6th | | | " | " |
| 6th | " | one 2 | " | dose | " | " | 5th | | | " | " |
| 7th | " | one 2 | " | " | " | " | 5th | | | " | " |
| 8th | " | one 2 | " | " | " | " | 4th | | | " | " |
| 9th | " | one 1 | " | " | " | " | 3d | | | " | " |
| 10th | " | one 2 | " | " | " | " | 5th | | | " | " |
| 11th | " | one 2 | " | " | " | " | 5th | | | " | " |
| 12th | " | one 2 | " | " | " | " | 4th | | | " | " |
| 13th | " | one 2 | " | " | " | " | 4th | | | " | " |
| 14th | " | one 2 | " | " | " | " | 3d | | | " | " |
| 15th | " | one 2 | " | " | " | " | 3d | | | " | " |

The "intensive" method is as follows:

| | | | | | | | |
|---|---|---|---|---|---|---|---|
| 1st day, | morning, | two 3 cc. | doses of | 14th | and | 13th-day | brains. |
| | evening, | two 3 cc. | " | 12th | " | 11th-day | " |
| 2d " | morning, | two 3 cc. | " | 10th | " | 9th-day | " |
| | evening, | two 3 cc. | " | 8th | " | 7th-day | " |
| 3d " | morning, | one 2 cc. | dose of | 6th-day | | | brain. |
| | evening, | one 2 cc. | " | 6th-day | | | " |

| | | | | | | | |
|---|---|---|---|---|---|---|---|
| 4th day | one | 2 cc. | dose | of the | 5th-day | brain. |
| 5th " | one | 2 " | " | " | 5th " | " |
| 6th " | one | 2 " | " | " | 4th " | " |
| 7th " | one | 1 " | " | " | 3d " | " |
| 8th " | one | 2 " | " | " | 4th " | " |
| 9th " | one | 1.5 " | " | " | 3d " | " |
| 10th " | one | 2 " | " | " | 5th " | " |
| 11th " | one | 2 " | " | " | 5th " | " |
| 12th " | one | 2 " | " | " | 4th " | " |
| 13th " | one | 2 " | " | " | 4th " | " |
| 14th " | one | 2 " | " | " | 3d " | " |
| 15th " | one | 2 " | " | " | 3d " | " |
| 16th " | one | 2 " | " | " | 5th " | " |
| 17th " | one | 2 " | " | " | 4th " | " |
| 18th " | one | 2 " | " | " | 3d " | " |
| 19th " | one | 2 " | " | " | 5th " | " |
| 20th " | one | 2 " | " | " | 4th " | " |
| 21st " | one | 2 " | " | " | 3d " | " |

The best evidence of the efficiency of these methods is the fact that in selected cases but 0.54 per cent. of the persons bitten and so treated have contracted rabies.

In 1889 Babes and Lepp[6] conceived the idea of transmitting conferred immunity from one animal to another by means of the blood. However, their success was not great in this line. Since then Tizzoni and Cetanni[6] have worked out a method of producing immunity by the blood-serum of an immunized rabbit. They found that this serum would sterilize an emulsion of a rabid cord in five hours, and then proceeded to experiment with living animals, and succeeded in producing immunity in a majority of cases.

They inject this as Pasteur injects the virus, subcutaneously. Their starting-point or standard was called "virus fixe," which was obtained by passing the virus from rabbit to rabbit until it reached its most potent form. They then prepared a series of weakened solutions by subjecting this material to the action of gastric juice; and, beginning with the weakest virus, they gradually increased its strength daily, or nearly so, by which method they could protect rabbits, dogs, and sheep. By continuing the process they succeeded in twenty days, after seventeen injections, in producing such a large amount of antirabic substance in the serum that, if injected twenty-four hours before the poison, even so small a proportion as 1 : 25,000 of serum to the body-weight would protect an animal. They also found that by injecting a little larger dose, with a rabific virus, or twenty-four hours after it, that the serum would protect the animal. They obtained a more powerful serum by revaccinating sheep, the injections being made during a course of twelve days, 0.25 gramme of the emulsified cord per kilogramme of the body-weight being injected daily. This process may be continued as often as required, so long as the interval between the courses is of sufficient length to allow the patient to keep in good condition. The series of vaccination may be repeated at intervals varying from two to five months.

This method has many advantages over Pasteur's treatment, as the serum can be dried and kept for a long time, and yet retain its antirabic properties. It can be sent by mail and used in the home, as it is readily soluble in distilled water or sterilized normal salt solution. However, great care must be exercised to prevent its becoming contaminated.

Again, Pasteur's method has an advantage over this one, for by his method the antitoxin is produced in the body, and therefore begets a permanent immunity, while by Tizzoni's method the antitoxin is introduced into the system, and is, of course, soon eliminated, causing only a temporary protection.

Sanitary Police. 1. Reduce the number of dogs by taxation, the authorities destroying all dogs that do not wear a license-tag or muzzle.

2. Quarantine for three months all dogs entering the country, unless accompanied by an official certificate that the country where they came from is free from rabies, as Australia, Argentine Republic, etc.

3. Compulsory killing or securely locking up, for at least six months, of all dogs or cats suspected of rabies, or that have been bitten by a supposed rabid animal. File sharp points of tusks.

4. Cause every dog in a community where rabies has prevailed within a year to wear a muzzle; all unmuzzled dogs to be shot by the authorities.

5. Destroy, as far as possible, all foxes, wolves, badgers, martens, skunks, and other vermin in a district known to have rabies.

6. Destroy by fire, or thoroughly disinfect, kennels, litter, blankets, collars, ropes, sticks, rags, dishes, or any utensils that have come in contact with a rabid animal. Disinfect wagons or any vehicle used to transfer such a cadaver. Disinfect or destroy by fire the stall or manger where a rabid animal has been confined. If it has been at pasture, do not allow other animals to enter such pasture for one week or ten days, as they might have or receive a laceration and become infected, although it is generally alleged that the blood and saliva are not virulent over twenty-four hours after leaving the body,[11] but in a dry state may retain the virulence for a long time.

7. Do not allow people to go barefooted around a vicinity where rabies is prevalent.

8. Furnish owners of dogs with a description of the premonitory symptoms of rabies, that they may at once kill or shut up and report any suspected case to the authorities.

9. Forbid the sale or consumption of any part of or the products of any suspected animal; this refers to meat, hides, tallow, and milk particularly.

10. If any domestic animal be suspected of rabies, shut it up, and, upon the appearance of the symptoms, destroy it.

11. Burn or bury deeply the cadavers of all victims, and do not allow them to be skinned.

In closing, I wish to acknowledge my gratefulness for the assistance received from Professors Law and Moore.

(To be continued.)

---

Dr. J. P. Turner, Inspector of Dairy-farms in the District of Columbia, reports the following interesting case: A grade Jersey cow was served by the bull sometime in December, 1897, and was considered as being pregnant, as she did not "bull" any more; was only served once. In March, 1898, the flow of milk ceased, and soon after she aborted a fœtus, which was about three months old. The flow of milk again returned, and the cow milked until the last of July. This cow gave birth to a large, well-formed heifer calf about September 25th.

Query. The same writer desires to hear from other veterinarians as to what percentage of fox-terrier puppies are born with short tails. He reports one litter of six pups, three of which had short tails.

---

Veterinarian E. C. Porter, of New Castle, Pa., is a hustler in politics. He was elected Coroner of Lawrence County on November 8th, by a plurality of 2254, running 650 ahead of the other Republican candidates. Among his stalwart supporters was Veterinarian E. E. Bittles.

Dr. R. S. Huidekoper, lieutenant-colonel of the First Army Corps, recently located at Porto Rico, is temporarily in Washington, D. C.

